# CooVar: Co-occurring variant analyzer

**DOI:** 10.1186/1756-0500-5-615

**Published:** 2012-11-01

**Authors:** Ismael A Vergara, Christian Frech, Nansheng Chen

**Affiliations:** 1 Department of Molecular Biology and Biochemistry, Simon Fraser University, 8888 University Drive, Burnaby, B.C., V5A 1S6, Canada; 2 GenomeDx Biosciences Inc, 1595 West 3rd Avenue, Vancouver, BC, V6J 1J8, Canada

**Keywords:** Variant effect prediction, Variant annotation, Genomic variation, Sequence analysis, Protein-coding transcript, Indel, SNV, Insertion, Deletion

## Abstract

**Background:**

Evaluating the impact of genomic variations (GV) on protein-coding transcripts is an important step in identifying variants of functional significance. Currently available programs for variant annotation depend on external databases or annotate multiple variants affecting the same transcript independently, which limits program use to organisms available in these databases or results in potentially incorrect or incomplete annotations.

**Findings:**

We have developed CooVar (Co-occurring Variant Analyzer), a database-independent program for assessing the impact of GVs on protein-coding transcripts. CooVar takes GVs, reference genome sequence, and protein-coding exons as input and provides annotated GVs and transcripts as output. Other than similar programs, CooVar considers the combined impact of all GVs affecting the same transcript, generating biologically more accurate annotations. CooVar is operated from the command-line and supports standard file formats VCF, GFF/GTF, and GVF, which makes it easy to integrate into existing computational pipelines. We have extensively tested CooVar on worm and human data sets and demonstrate that it generates correct annotations in only a short amount of time.

**Conclusions:**

CooVar is an easy-to-use and lightweight variant annotation tool that considers the combined impact of GVs on protein-coding transcripts. CooVar is freely available at http://genome.sfu.ca/projects/coovar/.

## Findings

### Introduction

One central goal of many genomics projects is to detect different types of genomic variations (GVs) and to understand how these GVs explain differences at the phenotypic level, for example, between healthy and diseased individuals [1,2]. Accurate and comprehensive detection of GVs, including single-nucleotide variations (SNVs), insertions and deletions, has been greatly facilitated by the development of next generation sequencing technologies [3] and variation detection methods [4]. After GVs are defined, evaluation of their functional impact on protein-coding transcripts becomes the primary focus. Many programs have been developed for this task, of which Ensembl’s Variant Effect Predictor (VEP) [5], GATK’s VariantAnnotator [6], Sequence Variant Analyzer (SVA) [7] and ANNOVAR [8] are among the more popular ones.

Current variant annotation programs have important limitations. First, they assess the effects of multiple co-occurring GVs on the same transcript independently, which can be problematic when nearby GVs alter each other’s effect [9]. For example, a small deletion can restore the open reading frame (ORF) disrupted by a small insertion co-occurring nearby on the same transcript. A second limitation is that most programs are tightly coupled to external databases, making their use inconvenient or even impractical for users who work on organisms whose genome sequence or annotation is not available in these databases.

### Implementation

We have developed an easy-to-use Perl program named CooVar (Co-occurring Variant Analyzer) to address these limitations. CooVar takes as input (i) a list of GVs in the popular Variant Call Format (VCF) [10] or in a simpler tab-delimited file format, (ii) the reference genomic DNA sequence in FASTA format, and (iii) protein-coding exon coordinates in GFF or GTF format.

The core output of CooVar are two files: a GVF file [11] reporting the functional impact of each input GV on transcripts, and a GFF file including (i) the transcript models, (ii) all GVs impacting each transcript, and (iii) a prediction of how GVs impact the function of each transcript. The functional impact of GVs on protein-coding transcripts is annotated as: ORF_INTACT, if the transcript is not impacted by any GVs; ORF_PRESERVED, if the transcript is impacted by GVs but these GVs do not introduce internal stop or splice site variants; ORF_DISRUPTED, if an internal stop or splice site variant is present; and FULLY_DELETED, if the transcript is deleted. In the case of a transcript that has its ORF disrupted by an internal stop codon, CooVar provides the percentage location of the first internal stop codon in the variant peptide compared to the reference.

CooVar classifies GVs according to the GVF v1.05 specification for structural variants described in the Sequence Ontology (SO) Project [12]. For SNVs, these categories include *silent_mutation*, *synonymous_codon*, *conservative_missense_codon*, *non_conservative_missense_codon*, *stop_gained*, *stop_lost*, *splice_acceptor_variant*, and *splice_donor_variant*. Insertions and deletions are classified into the categories *silent_mutation*, *frameshift_variant*, *inframe_variant*, *splice_acceptor_variant*, and *splice_donor_variant*. The functional impact of missense SNVs causing amino acid changes is further evaluated with the Grantham score [13] and annotated as *CONSERVATIVE* or *MODERATELY_CONSERVATIVE* (both classified as *conservative_missense_codon*) and *MODERATELY_RADICAL* or *RADICAL* (both classified as *non_conservative_missense_codon*) [14]. For SNVs impacting protein-coding exons, CooVar also reports both the amino acid change and the codon change between the reference genome and the variant. This allows the user to observe immediately if a change in a codon is caused by one, two or three co-occurring substitutions at the same codon. Furthermore, CooVar lists separately all those SNVs that fall into multiple categories by impacting two or more protein-coding transcripts differently (e.g. synonymous *vs*. missense).

In addition to the annotation of individual transcripts and GVs, CooVar outputs various summary statistics. For example, CooVar generates statistics on the codon bias for synonymous versus non-synonymous SNVs. In two other files CooVar outputs the length distribution of indels across the whole genome versus the length distribution of indels impacting only protein-coding transcripts as a way to detect biases towards non-frameshift indels in exonic regions. The file *variant.stat* provides information on the distribution of internal stop codons and on the total number of transcripts affected by SNVs, insertions and deletions, or by any combination of those. If the --*circos* flag is used, CooVar computes the genomic distribution of SNVs, insertions, deletions and coding exons in a format compatible with the Circos tool for visualization [15].

One advantage of CooVar over other programs is that it provides full-length variant transcript and protein sequences in FASTA format as output, which can be useful for downstream analyses (for example for sequence alignments). The same information is provided at the exon level in two additional files. Since a direct comparison between the reference and variant transcript is also desirable, CooVar provides an exon-based alignment of reference and variant sequences for each transcript, with variant nucleotides marked in uppercase. This makes it easy to spot all SNVs, insertions and deletions that impact a given protein-coding transcript in a region of interest.

Another commonly requested feature in variant annotation is to identify GVs that overlap with protein domains. This is because GVs affecting conserved domains are more likely to be of functional importance. With CooVar this analysis can be performed in two steps. First, the script *protein2genome.pl* can be used to map protein (domain) coordinates to the genome, which generates a GFF file with genomic coordinates. The script *annotate-regions.pl* can then be used to compute the overlap between this GFF file and the CooVar GVF output file. Overlap computation is performed efficiently using interval trees and generally finishes within a few minutes, even for very large data sets. The result of this two-step process is a new GVF file in which GVs are annotated with the protein domains they overlap with. It is worth mentioning that *annotate-regions.pl* script is generic and can also be used to annotate GVs that overlap with non-protein-coding regions (for example transcription factor binding sites) as long as coordinates for these regions are provided in the required input GFF format.

More detailed information about program parameters and input file formats can be found in the program README file or in the Perl scripts themselves.

### Results and discussion

**Figure 1:**
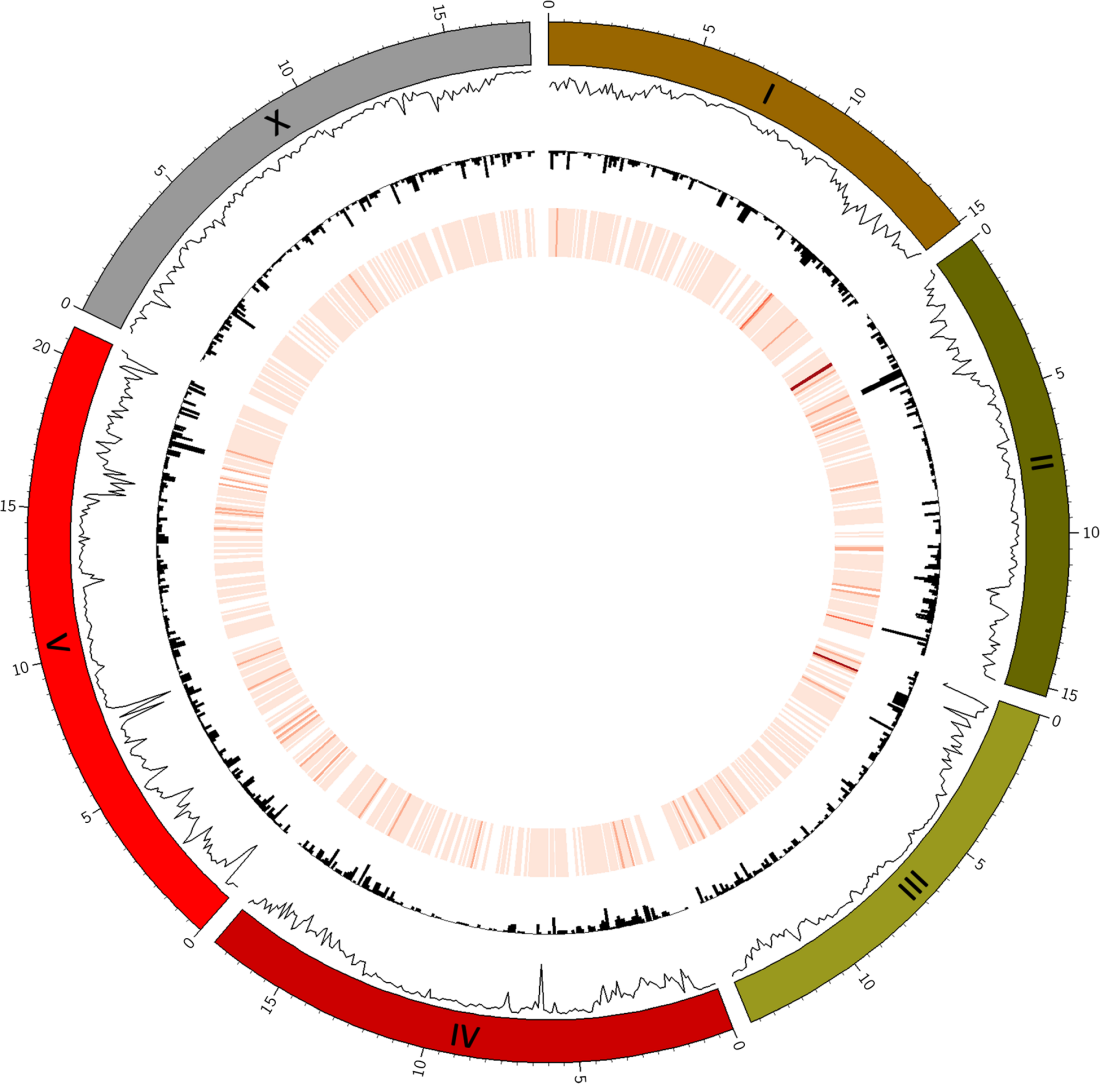
**Distribution of SNVs, insertions, and deletions along the *C. elegans *genome for Hawaiian isolate CB4856.** Segmented rings on the outside represent the six *C. elegans* chromosomes. Going from outside to inside, the line plot shows SNV density (inward pointing peaks = higher density), histograms represent the density of deletions (also drawn inwards), and the heatmap depicts the density of insertions (dark red = higher density) detected in the Hawaiian isolate CB4856. Note the generally higher density of SNVs towards the telomeres and the presence of chromosome-internal peaks on chromosome IV and V. Data points for this image were automatically generated by CooVar using the --*circos* option. Circos was then used to generate the image. Circos configuration files necessary to create this type of image are provided with the *C. elegans* test data set at http://genome.sfu.ca/projects/coovar/.

We have tested CooVar on two datasets, both of which are available from the project website. The first dataset corresponds to 120,638 GVs (116,999 SNVs, 1,553 insertions ranging from 1 to 34 bp in length and 2,086 deletions ranging from 1 to 24 bp in length) detected in the Hawaiian isolate CB4856 of the model organism *Caenorhabditis elegans*. CB4856 GVs, the N2 reference genome (isolated in Bristol, England) and 24,256 annotated N2 protein-coding transcript models were obtained from WormBase release WS210 [16]. CooVar had a processing time of 10 minutes for this data set and classified 15,293 transcripts as ORF_INTACT, 8,446 transcripts as ORF_PRESERVED, and 517 transcripts as ORF_DISRUPTED. Figure 1 shows a Circos image with the distribution of SNVs, deletions, and insertions along the six *C. elegans* chromosomes. This image was generated by using the CooVar output files (option --*circos*) as input for Circos (version 0.62-1) [15].

The second dataset contains 4,044,200 human GVs detected in an anonymous individual (HG00732-200-37-ASM) sequenced by Complete Genomics. This data set was recently made publicly available for the research community as part of a larger 69 genome data set [17,18]. HG00732-200-37-ASM variants were first extracted from the 69-sample VCF file using *vcf-subset*[10]. We then discarded all but the first alternative allele and used the filtered VCF file as input to CooVar. The genomic reference sequence and the protein-coding gene set were both obtained from the Ensembl web site (release GRCh37.68, hg19). For comparison, we annotated the exact same VCF file with Ensembl’s Variant Effect Predictor (VEP) [5]. VEP was run locally using the Perl script *variant_effect_predictor*.*pl* and configured to retrieve Ensembl data (release 68) over the internet (−−*host useastdb.ensembl.org*). The VEP output for the HG00732-200-37-ASM data set can be downloaded from the CooVar project homepage.

Results of this comparison are summarized in Table 1. CooVar took 36 minutes to process the complete human data set and reported 4,158,840 annotated variants. VEP outputted 4,043,939 variants and finished within 37 hours and 24 minutes. The overall increased number of variants reported by CooVar is because CooVar decomposes compound input VCF variants into sometimes multiple SNV and indel variants, which are then annotated and reported separately. For example, CooVar decomposes a VCF input variant with the reference allele “ATG” and the alternative allele “AC” into one SNV (T->C) and one deletion variant (with G being the deleted base in this case). In contrast, VEP will annotate such compound variants as a single variant, resulting in sometimes ambiguous or nonspecific classification results (see examples below).

**Table 1:** Comparison of GVs annotated with CooVar and VEP for human individual HG00732-200-37-ASM

	**CooVar**	**VEP**
Runtime	36m	37h 24m
Total reported GVs	4,158,840	4,043,939
Intronic/intergenic/UTR	4,133,885	4,019,490
Impacting protein-coding exon	24,955	24,449
Synonymous/stop retained	11,585	11,434
Missense	12,011	11,576
Conservative (%)^$^	9,526 (79.3)	7,447 (64.3)
Non-conservative (%)^+^	2,485 (20.7)	2,110 (18.3)
Unknown consequence (%)	0 (0)	2,019 (17.4)
Splice donor/acceptor	97	184
Stop lost	47	46
Stop gained	137	134
Frameshift	470	490
Inframe	199	165
Other	0	31
Multiple*	409	389
ORF disrupted	782^¥^	871^§^

Each GV reported by CooVar (v0.05) and VEP (v2.6) was assigned to one (and only one) of the above categories. $ CooVar: Grantham score *conservative* or *moderately conservative*; VEP: SIFT *benign*; + CooVar: Grantham score *moderately radical* or *radical*; VEP: SIFT *deleterious.* * GVs assigned to more than one category due to differential impact on different transcripts. ¥ Presence of internal stop codon within the first 70% of ORF length, after applying all variants. § Predicted frameshift or stop gain variant within the first 70% of ORF length. Abbreviations: GV … genomic variation; ORF … open reading frame; VEP… Variant Effect Predictor; UTR… untranslated region.

As expected, both programs classify the vast majority of variants as not impacting protein-coding exons (Table 1, category *intronic/intergenic/UTR*). Only 0.6% of all variants (24,955 variants by CooVar and 24,449 by VEP) are predicted to impact protein-coding exons in some form. To allow for a detailed comparison of annotation results, we assigned variants impacting protein-coding exons into one (and only one) of the following categories: variants not altering protein translation (*synonymous/stop retained*); variants altering protein translation (*missense*); variants impacting AG/GT splice site di-nucleotides (*splice donor/acceptor*); variants leading to stop codon loss (*stop lost*) or gain (*stop gained*); and insertions or deletions that shift (*frameshift*) or preserve the open reading frame (*inframe*). Thirty-one VEP variants could not be assigned to one of these categories and were classified as *other*. This includes variants that VEP nonspecifically annotated as *coding_sequence_variant*. A number of variants (409 for CooVar, 389 for VEP) could not be unambiguously assigned to a single category because they impact multiple transcripts differently and were classified as *multiple*.

Overall, we find that numbers of GVs in each category agree well between CooVar and VEP (Table 1). Both programs predict ~11,500 synonymous variants and about the same number of missense variants. CooVar’s Grantham score classifies ~20% of missense variants as *moderately radical* or *radical*, which agrees well with the VEP SIFT classification scheme that predicts 18% of missense variants to be *deleterious*. Both programs predict about 50 stop lost mutations and 135 stop gain mutations. Interestingly, VEP predicts 20 more frameshift variants than CooVar (490 vs. 470 variants) and 34 less inframe variants (165 vs. 199 variants). Also, the number of predicted splice site variants is markedly different between the two programs, with almost twice as many splice site variants predicted by VEP (184 variants) than CooVar (97 variants).

To understand the nature of these differences, we performed a detailed manual analysis of GVs that were differently annotated between CooVar and VEP. In general, we find that the main source of discrepancy between CooVar and VEP is due to the fact that CooVar but not VEP recognized the presence of SNVs within more complex or compound VCF input variants. For example, reference and alternative allele in the VCF input variant 11:11,292,688:GGGTCAGGACGCG->GGGTCAGGACGCC differ by only a single SNV (G->C, underlined). CooVar correctly reports this variant as synonymous SNV while VEP annotates it less specifically as *coding_sequence_variant,* without information on codon impact. The different numbers in stop lost and stop gained variants are attributable to the same effect. For example, CooVar interprets multi-SNV variant 19:43,922,549:AGA->TGC as stop lost variant while VEP annotates it as *coding_sequence_variant* and *3_prime_UTR_variant*. Manual inspection showed that the first of the three SNVs encoded by this input variant (A->T) indeed changes the stop codon of transcript ENST00000253435 from TAA to TAT, suggesting that the CooVar prediction is correct.

The much larger number of splice site variants predicted by VEP is also explained by the higher resolution with which CooVar decomposes complex VCF input variants. For example, variant 11:117,303,853:CCCAGT->CCCAGC is annotated as splice donor variant by VEP but not CooVar, which reports it as synonymous SNV. Manual inspection showed that the coordinates of this variant (117,303,853–117,303,858) indeed overlap with a donor splice site of transcript ENST00000527706, but the actual SNV encoded by this variant (T->C) is in fact synonymous. Thus, in this case, a simple coordinate overlap analysis as seems to be performed by VEP produces an incorrect result. Other examples of this type include variant 7:101,194,424:CGTAA->TGTAA (CooVar: synonymous), 5:159,835,654:TACCA->TACCG (CooVar: missense), or 19:16,612,363:GTG->GTA (CooVar: silent).

Complex input variants also account for discrepancies observed between indel annotations. We randomly picked and examined 10 of the 34 inframe variants predicted by CooVar but not VEP. For 9 out of these, we find that they are genuine inframe indels that VEP classified as missense (for example 10:126,715,151:TGCAGAGGAGC->TGCGGAGGAGCCGCAGGCTGGGGCTGCAGGGC or 12:53,045,625:CT->CCGCTGCCGCCTCCAAAGCC; note the length difference is a multiple of 3 in both cases). Why VEP classifies these variants as missense variants was not obvious to us. The remaining variant of these ten variants was actually classified as inframe variant by VEP but assigned to category *multiple* by our classification scheme because VEP predicts it as both inframe and stop gained variant.

Another main source of discrepancy in indel classification arose from so called “boundary indels”. We refer to boundary indels as indels that fall right next to the start or end of coding exons, thus leaving some uncertainty about the exact impact of these variants on the protein-coding transcript. Variant 7:142,494,013 is an example of an insertion where the exact placement of the inserted sequence is ambiguous, resulting in a predicted frameshift insertion by CooVar but in a predicted *coding_sequence_variant* and *5_prime_UTR_variant* by VEP. Most of the frameshift variants predicted by VEP but not CooVar represent boundary indels. Representative examples include 11:111,853,106:G->GC (1-bp insertion right before coding exon), 16:76,311,602:G->GT (1-bp insertion right after coding exon), 16:31,770,696:GA->GAA (1-bp insertion into start codon), and 17:39,254,335:AT->ATT (1-bp insertion before start codon). We manually inspected all 20 frameshift variants predicted by VEP but not CooVar and confirm that CooVar predictions appear to be correct, *i.e.* these variants are likely not causing frameshift mutations in affected transcripts.

We were also interested in the number of ORFs that were predicted to be disrupted by both CooVar and VEP. For this particular comparison, we defined a CooVar ORF as being disrupted if an internal stop codon occurred within the first 70% of the ORF’s length after applying all GVs to a transcript. CooVar provides the position of the first internal stop codon as part of its output. VEP does not provide ORF status information in its output, so we defined a VEP ORF as being disrupted if VEP predicted at least one frameshift or stop gain variant within the first 70% of the ORF’s length. Using these criteria, we find that CooVar predicts 782 ORFs to be disrupted while VEP predicts 871 ORFs as disrupted (Table 1). We inspected about half (48) of the transcripts that had assigned a different ORF status by the two programs and found that most of them (20 ORFs, *e.g.* transcript ENST00000376343) carry a frame-shifting indel that does not introduce an internal stop codon albeit it changes the translated protein sequence downstream. Thus, although for these transcripts a significant portion of the ORF (>30%) is changed in terms of its protein sequence, the length of the ORF remains intact. Seventeen of the 48 inspected transcripts (*e.g.* ENST00000222270) had a frameshift predicted by VEP but not CooVar due to boundary indels as discussed above. Five of the 48 transcripts had already internal stop codons in the reference sequence and hence were not annotated as disrupted by CooVar.

**Figure 2:**
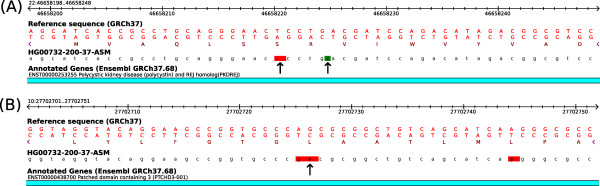
**GVs affecting the same protein-coding transcript must be assessed together to correctly predict their functional impact.** Panel **A** shows an example where two neighboring frameshift indels (1-bp insertion and 1-bp deletion, indicated by arrows) cancel each others effect, restoring the original ORF. Panel **B** shows an example where an otherwise synonymous SNV (G->A, indicated by arrow) causes a missense mutation due to the effect of a neighboring SNV (A->G). In both panels, the first three rows show the reference nucleotide sequence on the forward strand, the reference nucleotide sequence from the reverse strand, and the reference protein sequence translation from the annotated ORF. Note that both ORFs are encoded on the reverse strand, so sequences must be read from right to left. The track below shows the variant sequence detected in human individual HG00732-200-37-ASM, with critical GVs highlighted by arrows. The blue horizontal bar represents the Ensembl protein-coding transcript spanning this genomic region.

Most importantly, the remaining six ORFs predicted to be disrupted by VEP but not CooVar carry neighboring indels that cancel each others effect, restoring the open reading frame. Figure 2A shows one such example affecting Ensembl transcript ENST00000253255. This transcript carries a 1bp insertion at position 22:46,658,224 and a nearby 1bp deletion at position 22:46,658,220. When evaluated independently, the impact of the insertion and the deletion on this transcript is a frameshift mutation, disrupting the ORF. But when evaluating the joint effect that these GVs have on the transcript it results in a preserved ORF, as reported by CooVar. A similar issue arises from co-occurring SNVs. In the example shown in Figure 2B, VEP classifies SNV 10:27,702,726:G->A as synonymous because it changes codon CTG on the reverse strand (coding for leucine) to TTG (also coding for leucine). However, CooVar considers the combined effect of this SNV and a neighboring SNV (10:27,702,725:A->G) that affects the same codon. When evaluated together, 10:27702726:G->A is recognized by CooVar as missense SNV that changes the codon from CTG to TCG, which codes for amino acid serine. These two examples illustrate the importance of assessing the impact of co-occurring GVs together to correctly judge their functional impact [9].

We conclude that CooVar is a fast and light-weight alternative to currently existing variant annotation tools that is particularly useful for non-model organisms. CooVar produces very similar results as other popular tools, but, under certain circumstances, generates biologically more accurate annotations by considering the combined effect of co-occurring GVs on protein-coding transcripts.

## Availability and requirements

**Project name**: CooVar: Co-occurring Variant Analyzer

**Project home page**: http://genome.sfu.ca/projects/coovar

**Operating system(s)**: Windows, Linux, Mac OS-X

**Programming language**: Perl 5.8.8

**Other requirements**: The following Perl modules are required by CooVar and need to be installed: Cwd, Getopt::Long, POSIX, File::Basename, List::Util, Bio::DB::Fasta, Bio::Seq, Bio::SeqUtils, Bio::SeqIO, Set::IntervalTree, Set::IntSpan

**License**: GNU GPL

**Any restrictions to use by non-academics**: none

The latest version of the program can be obtained from the project webpage. CooVar version 0.05 is included as online supplementary material (Additional file 1).

## Abbreviations

GV: Genomic Variation; GFF: Generic Feature Format; GTF: Gene Transfer Format; SNV: Single Nucleotide Variant; Indel: Insertion or deletion; GVF: Genomic Variant Format; ORF: Open Reading Frame; VCF: Variant Call Format.

## Competing interests

The authors declare that they have no competing interest.

## Authors’ contribution

IAV and NC conceived the study. IAV developed the method and original program. CF co-developed and tested the program. IAV, CF, and NC wrote the manuscript. All authors read and approved the final manuscript.

## Authors’ information

Ismael A Vergara and Christian Frech were joint first author.

## Supplementary Material

Additional file 1 CooVar program tarball (version 0.05), including README and test scripts. Click here for file

## References

[B1] MacArthur DG Tyler-Smith C Loss-of-function variants in the genomes of healthy humans Hum Mol Genet 2010 19 R2 R125 R130 10.1093/hmg/ddq365 20805107 PMC2953739

[B2] Stankiewicz P Lupski JR Structural variation in the human genome and its role in disease Annu Rev Med 2010 61 437 455 10.1146/annurev-med-100708-204735 20059347

[B3] Shendure J Ji H Next-generation DNA sequencing Nat Biotechnol 2008 26 10 1135 1145 10.1038/nbt1486 18846087

[B4] Medvedev P Stanciu M Brudno M Computational methods for discovering structural variation with next-generation sequencing Nat Methods 2009 6 11 Suppl S13 S20 19844226 10.1038/nmeth.1374

[B5] McLaren W Pritchard B Rios D Chen Y Flicek P Cunningham F Deriving the consequences of genomic variants with the Ensembl API and SNP Effect Predictor Bioinformatics 2010 26 16 2069 2070 10.1093/bioinformatics/btq330 20562413 PMC2916720

[B6] McKenna A Hanna M Banks E Sivachenko A Cibulskis K Kernytsky A Garimella K Altshuler D Gabriel S Daly M The Genome Analysis Toolkit: a MapReduce framework for analyzing next-generation DNA sequencing data Genome Res 2010 20 9 1297 1303 10.1101/gr.107524.110 20644199 PMC2928508

[B7] Ge D Ruzzo EK Shianna KV He M Pelak K Heinzen EL Need AC Cirulli ET Maia JM Dickson SP SVA: software for annotating and visualizing sequenced human genomes Bioinformatics 2011 27 14 1998 2000 10.1093/bioinformatics/btr317 21624899 PMC3129530

[B8] Wang K Li M Hakonarson H ANNOVAR: functional annotation of genetic variants from high-throughput sequencing data Nucleic Acids Res 2010 38 16 e164 10.1093/nar/gkq603 20601685 PMC2938201

[B9] MacArthur DG Balasubramanian S Frankish A Huang N Morris J Walter K Jostins L Habegger L Pickrell JK Montgomery SB A systematic survey of loss-of-function variants in human protein-coding genes Science 2012 335 6070 823 828 10.1126/science.1215040 22344438 PMC3299548

[B10] Danecek P Auton A Abecasis G Albers CA Banks E DePristo MA Handsaker RE Lunter G Marth GT Sherry ST The variant call format and VCFtools Bioinformatics 2011 27 15 2156 2158 10.1093/bioinformatics/btr330 21653522 PMC3137218

[B11] Reese MG Moore B Batchelor C Salas F Cunningham F Marth GT Stein L Flicek P Yandell M Eilbeck K A standard variation file format for human genome sequences Genome Biol 2010 11 8 R88 10.1186/gb-2010-11-8-r88 20796305 PMC2945790

[B12] Eilbeck K Lewis SE Mungall CJ Yandell M Stein L Durbin R Ashburner M The Sequence Ontology: a tool for the unification of genome annotations Genome Biol 2005 6 5 R44 10.1186/gb-2005-6-5-r44 15892872 PMC1175956

[B13] Grantham R Amino acid difference formula to help explain protein evolution Science 1974 185 4154 862 864 10.1126/science.185.4154.862 4843792

[B14] Li WH Wu CI Luo CC Nonrandomness of point mutation as reflected in nucleotide substitutions in pseudogenes and its evolutionary implications J Mol Evol 1984 21 1 58 71 10.1007/BF02100628 6442359

[B15] Krzywinski M Schein J Birol I Connors J Gascoyne R Horsman D Jones SJ Marra MA Circos: an information aesthetic for comparative genomics Genome Res 2009 19 9 1639 1645 10.1101/gr.092759.109 19541911 PMC2752132

[B16] Harris TW Antoshechkin I Bieri T Blasiar D Chan J Chen WJ De La Cruz N Davis P Duesbury M Fang R WormBase: a comprehensive resource for nematode research Nucleic Acids Res 2010 38 Database issue D463 D467 19910365 10.1093/nar/gkp952 PMC2808986

[B17] Drmanac R Sparks AB Callow MJ Halpern AL Burns NL Kermani BG Carnevali P Nazarenko I Nilsen GB Yeung G Human genome sequencing using unchained base reads on self-assembling DNA nanoarrays Science 2010 327 5961 78 81 10.1126/science.1181498 19892942

[B18] Complete Genomics 69 Genomes Data ftp://ftp2.completegenomics.com/Multigenome_summaries/Complete_Public_Genomes_69genomes_B37_mkvcf.vcf.bz2

