# Supplementary figures and images for: CooVar: Co-occurring variant analyzer
Source: BMC Res Notes. 2012 Nov 1;5:615. doi: 10.1186/1756-0500-5-615 (PMC3532326; doi:10.1186/1756-0500-5-615)

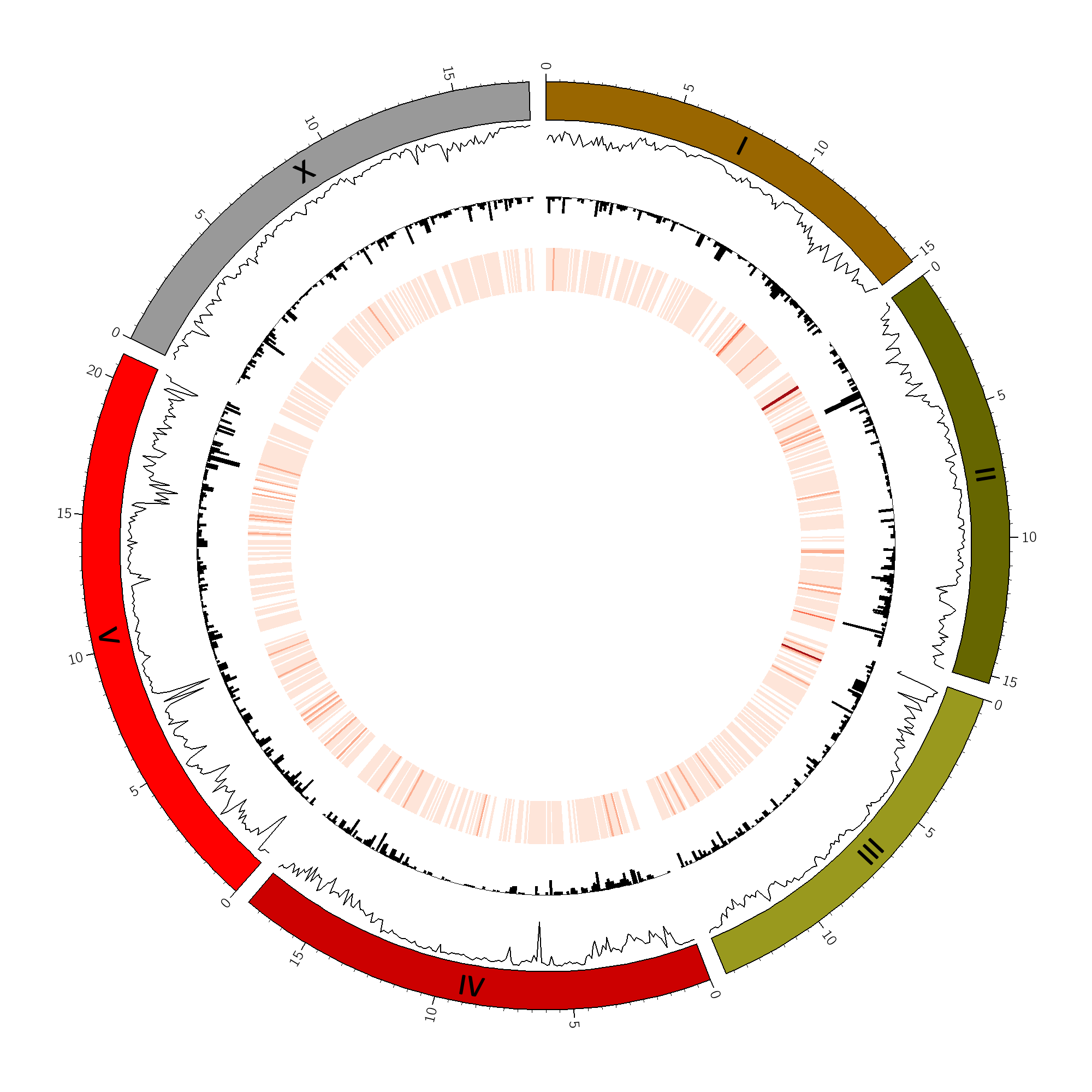

Supplement: Additional file 1 — CooVar program tarball (version 0.05), including README and test scripts. [file 1756-0500-5-615-S1.gz › coovar-0.05/test/celegans_CB4856/circos/elegans.circos.png]
